# Identification of genetic risk variants for deep vein thrombosis by multiplexed next-generation sequencing of 186 hemostatic/pro-inflammatory genes

**DOI:** 10.1186/1755-8794-5-7

**Published:** 2012-02-21

**Authors:** Luca A Lotta, Mark Wang, Jin Yu, Ida Martinelli, Fuli Yu, Serena M Passamonti, Dario Consonni, Emanuela Pappalardo, Marzia Menegatti, Steven E Scherer, Lora L Lewis, Humeira Akbar, Yuanqing Wu, Matthew N Bainbridge, Donna M Muzny, Pier M Mannucci, Richard A Gibbs, Flora Peyvandi

**Affiliations:** 1 Angelo Bianchi Bonomi Hemophilia and Thrombosis Center, U.O.S. Dipartimentale per la Diagnosi e la Terapia delle Coagulopatie, Fondazione IRCCS Cà Granda - Ospedale Maggiore Policlinico, Università degli Studi di Milano and Luigi Villa Foundation, Milan, Italy; 2 Human Genome Sequencing Center, Baylor College of Medicine, Houston, TX, USA; 3 Unit of Epidemiology, Fondazione IRCCS Cà Granda - Ospedale Maggiore Policlinico, Università degli Studi di Milano, Milan, Italy

**Keywords:** Deep vein thrombosis, venous thromboembolism, next-generation sequencing, target capture, multiplexing, FGA, rs6025, heamostateome, DVT, VTE

## Abstract

**Background:**

Next-generation DNA sequencing is opening new avenues for genetic association studies in common diseases that, like deep vein thrombosis (DVT), have a strong genetic predisposition still largely unexplained by currently identified risk variants. In order to develop sequencing and analytical pipelines for the application of next-generation sequencing to complex diseases, we conducted a pilot study sequencing the coding area of 186 hemostatic/proinflammatory genes in 10 Italian cases of idiopathic DVT and 12 healthy controls.

**Results:**

A molecular-barcoding strategy was used to multiplex DNA target capture and sequencing, while retaining individual sequence information. Genomic libraries with barcode sequence-tags were pooled (in pools of 8 or 16 samples) and enriched for target DNA sequences. Sequencing was performed on ABI SOLiD-4 platforms. We produced > 12 gigabases of raw sequence data to sequence at high coverage (average: 42X) the 700-kilobase target area in 22 individuals. A total of 1876 high-quality genetic variants were identified (1778 single nucleotide substitutions and 98 insertions/deletions). Annotation on databases of genetic variation and human disease mutations revealed several novel, potentially deleterious mutations. We tested 576 common variants in a case-control association analysis, carrying the top-5 associations over to replication in up to 719 DVT cases and 719 controls. We also conducted an analysis of the burden of nonsynonymous variants in coagulation factor and anticoagulant genes. We found an excess of rare missense mutations in anticoagulant genes in DVT cases compared to controls and an association for a missense polymorphism of *FGA *(rs6050; p = 1.9 × 10^-5^, OR 1.45; 95% CI, 1.22-1.72; after replication in > 1400 individuals).

**Conclusions:**

We implemented a barcode-based strategy to efficiently multiplex sequencing of hundreds of candidate genes in several individuals. In the relatively small dataset of our pilot study we were able to identify bona fide associations with DVT. Our study illustrates the potential of next-generation sequencing for the discovery of genetic variation predisposing to complex diseases.

## Background

Deep vein thrombosis (DVT) of the lower extremities, a common thrombotic disease often complicated by acute pulmonary embolism [1], has a strong genetic component as established by family [2-4] and twin studies [5], with a 3-fold increase in disease risk for siblings of individuals with DVT [2] and an estimated hereditary component of 60% [3]. Genetic risk factors include rare mutations in *PROC*, *PROS1 *and *SERPINC1 *leading to the deficiencies of natural anticoagulant proteins (protein C, protein S and antithrombin, respectively) and single nucleotide polymorphisms (SNPs) of *F5 *(rs6025 or factor V Leiden [FVL]) and of *F2 *(rs1799963 or prothrombin G20210A) [6]. More recently, genome-wide association studies (GWAS) identified associations of common SNPs at 6 different genomic loci (*CYP4V2, SERPINC1, GP6, F5, ABO *and *HIVEP1*) [7-9]. In spite of these recent observations, however, genetic variants established to influence the risk for DVT explain only a fraction of disease heritability [10].

Next-generation DNA sequencing is opening new avenues for genetic association studies in complex diseases, enabling to sequence large fractions of the human genome (or even the entire genome) at unprecedented speed and per-base costs. Re-sequencing the exome (i.e. the protein coding area of the genome) or the entire genome is becoming the gold standard for the identification of disease-causing mutations in Mendelian diseases [11-13]. However, the application of these techniques to common diseases is still limited by the high costs and considerable computational burden associated with the analysis of numerous samples. Sequencing of a few hundred genes or genomic loci is a much less expensive alternative to whole-genome (or exome) sequencing, suitable for the analysis of areas of the genome that are deemed to have particular relevance in the pathophysiology of a given disease. However, this regional sequencing approach, which requires sample multiplexing in order to achieve efficiency, is technically challenging. Multiplexing on next-generation sequencing platforms entails the use of DNA pools, which is affected by limitations in downstream genetic association analysis. These limitations include (a) loss of sensitivity to detect rare genetic variants, (b) uncertainty in allele frequency estimations determined by the unequal representation of samples within the pool, and (c) loss of individual sequence data hampering direct genotype-phenotype associations.

In this pilot study, we used genomic libraries with barcode sequence-tags as a means to overcome the limitations of pooling. Using this approach we were able to multiplex DNA-target capture and SOLiD sequencing while retaining individual sequence information. In what is one of the first practical applications of this technique, we sequenced the coding area of 186 genes involved in blood hemostasis/inflammation (i.e. two pivotal pathophysiological mechanisms of DVT) in 10 Italian patients with early-onset idiopathic DVT and 12 thrombosis-free controls. The goal of this pilot study was to develop pipelines for the application of next-generation sequencing in the setting of DVT and to test different analytical approaches for the identification of disease-associated variants.

## Methods

### Patients

Cases of DVT were selected from 2139 unrelated patients referred to the Angelo Bianchi Bonomi Hemophilia and Thrombosis Center, Milan, (Italy) for diagnostic workout and thrombophilia testing after a first episode of DVT of the lower limbs in the years 1995-2010. Patients were asked to bring to the center the diagnostic documentation of their thrombotic episodes and underwent a clinical interview. DVT had been diagnosed by compression ultrasonography or venography. All patients underwent a complete thrombophilia screening, including measurement of natural anticoagulant proteins, genotyping of FVL and prothrombin G20210A and search for antiphospholipid autoantibodies. Coagulation factor VIII and fibrinogen coagulant activities were also measured.

A patient selection flow-chart is shown in Additional file 1 Figure S1. Patients selected for next-generation sequencing were required to have (a) history of idiopathic DVT of the lower limbs, (b) age of disease onset < 55 years, (c) wild-type FVL and prothrombin G20210A genotypes, (d) absence of natural anticoagulant deficiencies, (e) negative search for anti-phospholipid autoantibodies, (f) been born in Lombardy, the 10 million people region of Italy that has Milan as a regional capital. Since 42 patients matched these criteria, patients were further prioritized based on age of onset, familial history of venous or arterial thrombosis, completeness of clinical information, DNA amount and quality. Patients included in the replication were all the remaining 719 patients with a first episode of idiopathic lower-limb DVT and available DNA. Controls included in the study were selected from a total of 1938 healthy Italian individuals recruited among friends and non-consanguineous relatives who accompanied patients to the Hemophilia and Thrombosis Center and agreed to be tested for thrombophilia. Previous arterial or venous thrombosis in the controls was excluded using a validated questionnaire [14]. Controls had similar age of the idiopathic DVT cases (± 5 years) and the same gender. Controls who underwent next-generation sequencing were matched with the patients for geographic provenience (born in Lombardy), in order to minimize population stratification. In case more than one control matched the same patient, control inclusion was random. The study was approved by the Institutional Review Board of the Fondazione IRCCS Ca' Granda - Ospedale Maggiore Policlinico and all subjects gave their informed consent. Patient recruitment, sampling and thrombophilia screening were carried out at the Angelo Bianchi Bonomi Hemophilia and Thrombosis Center, Milan (Italy). Next-generation DNA analysis and replication PCR and Sanger sequencing and associated analyses were carried out at the Human Genome Sequencing Center (HGSC), Baylor College of Medicine, Houston (USA).

### Target area selection

The protein coding exons, 3' and 5' UTRs, and the intron-exon boundaries of 186 genes were chosen as target area. Target genes included all coagulation factor genes, anticoagulant genes and genes involved in fibrinolysis, platelet adhesion and aggregation, cell-cell interaction, endothelial activation and inflammation. The genomic coordinate intervals corresponding to the target area were obtained from the UCSC Genome Browser database and sent to NimbleGen for probe design. Probes were designed for all of the submitted intervals, with slightly different genomic coordinates (i.e. tiled regions). The final target area spanned 644, 472 bp. Probes were arrayed on Roche NimbleGen HD2 2.1 M-probe custom chips. The complete lists of target and tiled coordinate-intervals and of the target genes are in the Additional file 2 and Additional file 1 Table S1.

### Experimental design

Two experiments were carried out. In the first experiment, genomic libraries from 4 DVT patients (DVT_P_01, DVT_P_02, DVT_P_03, DVT_P_04) and 4 controls (DVT_C_01, DVT_C_02, DVT_C_03, DVT_C_04) were captured on the same Roche NimbleGen HD2 chip and sequenced in the same ABI SOLiD 4 spot (one quarter of a slide). In the second, libraries from 8 cases (DVT_P_01, DVT_P_05, DVT_P_06, DVT_P_07, DVT_P_08, DVT_P_09, DVT_P_10, DVT_P_11) and 8 controls (DVT_C_05, DVT_C_06, DVT_C_07, DVT_C_08, DVT_C_09, DVT_C_10, DVT_C_11, DVT_C_12) were captured on one Roche NimbleGen HD2 chip and sequenced in two ABI SOLiD 4 spots. Sample DVT_P_11 failed capture during the second experiment. Patient DVT_P_01 was sequenced twice as a quality control procedure.

### Genomic library preparation, barcoding and enrichment of target DNA sequences

Two micrograms of genomic DNA were used to prepare libraries of DNA fragments that were ligated with ABI SOLiD P1-and P2-adaptors [15]. Different modified P2-adaptors, each containing a specific DNA-tag sequence (molecular barcode), were used for each individual library. Libraries with barcodes were used to prepare equimolar DNA pools of 8 and 16 samples. Four micrograms of DNA from each pool were hybridized on one Roche NimbleGen HD2 chip.

### ABI SOLiD 4 sequencing

Capture products underwent emulsion PCR with P1-adaptor mediated attachment of clonally-amplified templates to loading beads. Beads were covalently attached on glass slides at the P2-adaptor extremity. Sequencing by oligonucleotide ligation and detection (SOLiD) was performed on ABI SOLiD 4 platforms [15].

### Read barcode assignment and mapping to the reference genome

Reads with the barcodes were assigned to the corresponding sample using custom Perl scripts and mapped to reference human genome, NCBI36/hg18, using BFAST software [16]. Reads that mapped on the same starting and end coordinates, considered likely to be PCR duplicates, were marked in the binary alignment/mapping (BAM) files, where mapping information was stored.

### Genetic variant calls and quality control (QC)

Sorted BAM files were processed in a variant-calling pipeline consisting of a BAM filtering process and a variant calling process. In the first step, duplicate reads were eliminated from the BAM files, retaining only the read with the top mapping quality at each pair of start and end mapping coordinates. Also, reads with mapping quality score of less than 50 were expunged from the BAM files. In the variant-calling step, Samtools [17] was used to generate PILEUP files with read information at sites where mismatches from the reference sequence were detected. Consensus in the presence of mismatches, read- and base-quality parameters were used as criteria to distinguish genetic variants from sequencing errors, filtering high-quality calls. In a final QC process, this set of calls was further cleared from variants with an allele balance (variant allele reads/total number of reference plus variant allele reads) below 20% and/or with significant strand bias (i.e. sites at which the variant sequence did not appear on both forward and reverse reads) in spite of high variant quality.

### Variant annotation

Genetic variants were annotated on RefSeq database, dbSNP129 and 1000 Genomes pilot release (March 2010) [18] using Annovar software [19]. Missense variants were also annotated on SIFT [20] and Polyphen 2 [21].

### Association analysis

Several pieces of software have been developed to analyze genotyping result of commercially SNP-genotyping arrays (e.g. those used for GWAS), in particular the successful PLINK package [22]. On the other hand, few tools are available for genetic association analysis of next-generation sequencing datasets. For this reason we developed Nxtgen2plink.rb, a software capable of generating PLINK-compatible input files with phenotypic information on sequenced individuals and genotype calls at each site that was found to be variable in at least one of the sequenced individuals. The software uses read alignment and coverage information (BAM files), genetic-variant calls (PILEUP files) and quality control information on variants with low quality (also in the PILEUP file format) to generate genotype calls across all individuals. Details of the workflow of the Nxtgen2plink.rb software are presented in Additional file 1 Figure S2. Association analysis was the carried out by PLINK and custom Perl and Python scripts.

In our dataset we chose to carry out (a) association analysis by Fisher's exact test of all identified variants that had at least a minor allele frequency (MAF) of 8% in the entire dataset of cases plus controls (i.e. the threshold MAF enabling a variant to reach a statistical significance with p < 0.05 given the sample size) and a genotype missingness of less than 10% and (b) 'collapsing' analysis comparing the total number of nonsynonymous single nucleotide variants (SNVs) in coagulation factor and anticoagulant protein genes in DVT cases and controls.

### Replication

We chose to carry over to replication in up to 719 idiopathic DVT patients and 719 matched controls the top-5 variants from the single-variant association analysis. Replication was carried out by PCR and Sanger sequencing, which were chosen as replication techniques given the high-throughput capacity of HGSC, their accuracy and their potential to reveal neighboring genetic variation in linkage disequilibrium with or with similar effect to that of the variant undergoing replication. Genotype calls were performed automatically by SNP-detector [23] and 10% of the calls were verified manually on chromatograms. Replication comprised two stages (1) initial replication in 284 individuals (2) full replication in 1438 individuals. We selected for stage 2 variants that given their allele frequency and effect size estimated in stage 1 had 80% chances to be replicated at p < 0.005 in the entire cohort of 1438 individuals. Genetic association was carried out by PLINK. The interaction of rs6050 with FVL and prothrombin G20210A was tested using PLINK with the *epistasis *option. Association of rs6050 with DVT after adjustment for covariates was assessed by multivariable logistic regression using STATA 10 software.

## Results

### Patient selection and characteristics

After exclusion of patients who were non-Caucasian, refused to give consent, had insufficient DNA amount or low DNA quality (n = 374), 1765 patients with lower limb DVT were included in the selection of idiopathic cases for the study. Of these, 719 patients with idiopathic DVT were further selected for the replication stage of this study and another 11 patients were chosen for next-generation sequencing after prioritizing the list of the 42 eligible patients selected on the basis of pre-specified criteria (see 'Patients' in the Methods section). Of 1938 thrombosis-free Caucasian individuals recruited at the Hemophilia and Thrombosis Center in the same period, 1703 who had available DNA were included in the selection of controls. Twelve controls underwent next-generation sequencing and 719 participated to the replication stage of the study. The individual demographic, clinical and laboratory features of the patients and controls who underwent next-generation sequencing are in Additional file 1 Table S2. Table 1 reports the characteristics of the 1438 individuals that participated to the replication stage.

**Table 1:** Characteristics of the individuals included in the replication stages of the study.

	PATIENTS WITH IDIOPATHIC DVT	CONTROLS
n=	719	719

GEOGRAPHIC ORIGIN, n (%)		

*Lombardy*	453 (63)	490 (67)

*Northern Italy other than Lombardy*	89 (13)	84 (12)

*Central Italy*	30 (4)	20 (3)

*Southern Italy and islands*	109 (15)	107 (15)

*Not available*	38 (5)	18 (3)

MALE GENDER, n (%)	325 (45)	325 (45)

AGE, mean years (standard deviation)	42.5 (15)	42.5 (14)

PULMONARY EMBOLISM, n (%)	148 (20)	/

REFERRED FOR MORE THAN ONE EPISODE, n (%)	167 (23)	/

BODY MASS INDEX, kg/m^2^	25.9 (4.7)	24 (4)

FACTOR V LEIDEN (rs6025) GENOTYPE, n (%)		

*GG*	611 (85)	696 (97)

*AG*	102 (14)	23 (3)

*AA*	6 (1)	0

PROTHROMBIN G20210A (rs1799963) GENOTYPE, n (%)		

*GG*	654 (91)	695 (97)

*AG*	63 (8)	24 (3)

*AA*	2 (1)	0

NATURAL ANTICOAGULANT DEFICIENCIES, n (%)		

*Antithrombin*	22 (3)	2 (0.2)

*Protein C*	26 (3)	2 (0.2)

*Protein S*	30 (4)	10 (1)

LABORATORY MEASUREMENTS, mean (standard deviation)		

*Antithrombin,% *	97 (12)	99 (15)

*Protein C, %*	102 (27)	105 (22)

*Protein S, %*	105 (30)	111 (31)

*Prothrombin time, international normalized ratio*	1 (0.16)	0.99 (0.15)

*Activated partial thromboplastin time, ratio*	1 (0.1)	1 (0.1)

*Coagulation factor VIII coagulant activity, %*	142 (39)	114 (30)

*Fibrinogen coagulant activity, %*	304 (72)	291 (63)

### Sequencing statistics

To sequence the 700-kilobase target area at an average depth of coverage of 42X (after removal of duplicate reads) in 22 samples, 12,040,000,000 bp and ~240 million reads of raw sequence data were generated. Reads were mapped on the human reference genome, NCBI36/hg18, and non-duplicate reads were retained and used for genotype calls. An average of 7% of the reads mapped to the target region, corresponding to an enrichment of more than 300-fold (the target area constitutes 0.02% of the 3-gigabase human genome). On average, 98, 4% of the target was covered at least once, 91% of the target area had at least the 10X coverage required for confident variant calls and 80% had coverage > 20X indicating homogeneous high coverage of the target area. Coverage statistics and representative histograms can be found in the Additional file 1 (Table S3 and S4; Figure S3).

### Type and frequency of identified genetic variants

**Figure 1:**
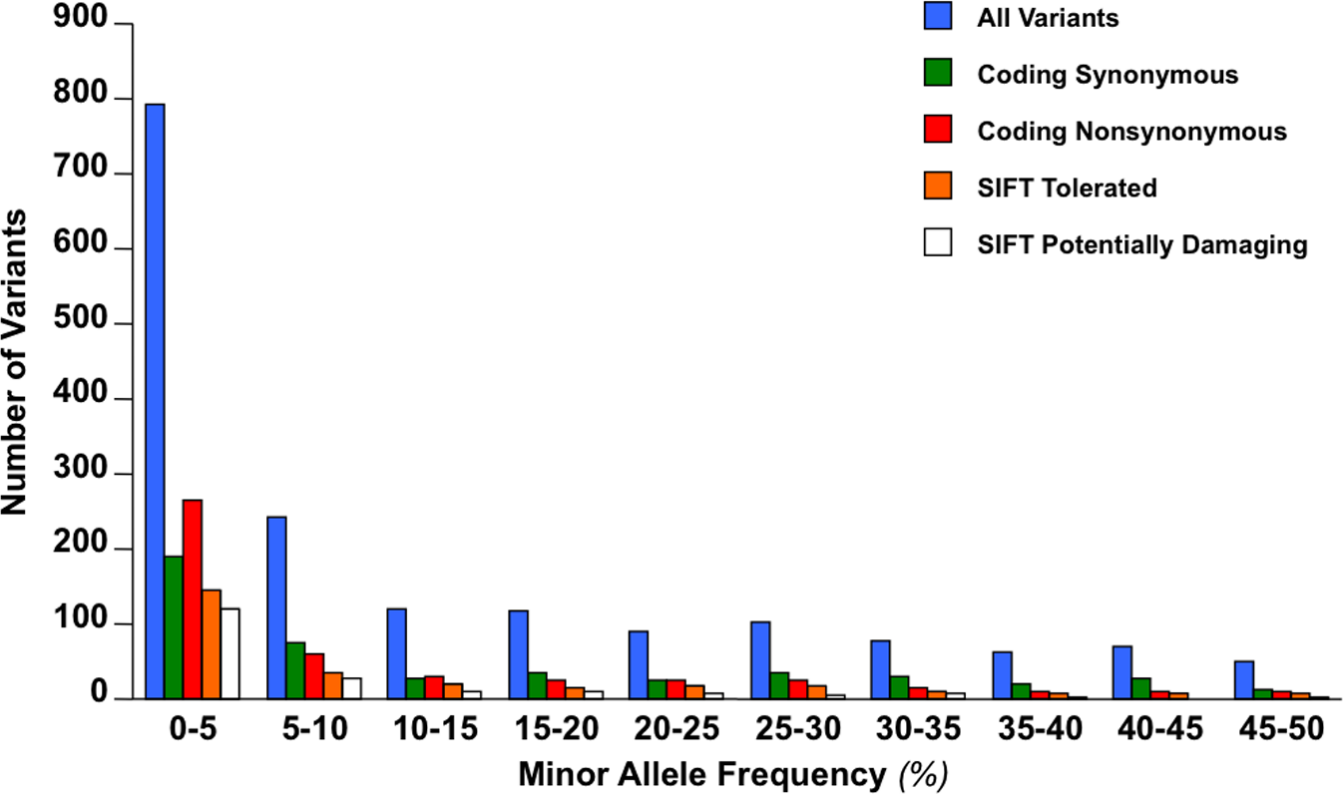
**Allele frequency distribution of single nucleotide variants with different functional annotation**.

In the 22 samples, 458 SNVs and 8 indels per individual were identified in the target region. Additional file 1 Tables S5-S7 show the characteristics and annotation of identified SNVs and indels. A total of 1778 high-quality SNVs were identified. Of these, 487 were coding nonsynonymous SNVs, which included 102 SNVs not present in dbSNP129 and 1000 Genomes pilot release. A total of 5 different nonsense variants were found (p.G2969X and p.G3170X in *COL6A3*; p.R412X in *KNG1*; p.R88X in *SERPINA10 *and p.R615X in *ZNF544*). Ninety-eight indels were called. The most frequent indel was an intronic, single-nucleotide deletion in *PLCG2 *(chromosome 16, coordinate 80461934). Figure 1 shows the frequency distribution of identified variants. Consistent with previous observations [11,18], the frequency interval between one observation and 5% yielded the highest number of variants, the highest nonsynonymous/synonymous ratio and the highest SIFT-predicted-damaging/SIFT-predicted-benign ratio. To verify the validity of variant calls and QC filters we (a) repeated the sequencing of one of the samples (DVT_P_01) (b) sequenced by PCR and Sanger all 22 samples at 35 sites where a SNV was found (c) verified by Pyrosequencing 19 genotypes at sites with variants that did not pass QC. Of 425 SNVs identified after QC in DVT_P_01 at the second sequencing experiment, 405 (95.3%) had been identified also in the first sequencing run of the same sample, showing the high reproducibility of next-generation sequencing. Of 765 genotypes available from both SOLiD and Sanger sequencing, 99.8% (697/698) of homozygous wild-type genotypes and 97% (65/67) of either heterozygous or homozygous variant genotypes were in agreement. None of the variants that did not pass QC was validated by Pyrosequencing.

### Disease-associated variants in the target genes

In order to search for the presence of disease-associated genetic variants, all identified SNVs were annotated on the human gene mutation database (HGMD^®^), a database that includes published mutations responsible for human diseases [24]. A total of 27 variants were present in HGMD^® ^and are listed in Additional file 1 Table S8. Of these variants, 9 had been reported in association with thrombotic diseases (either venous or arterial thrombosis) and 7 in association with DVT or DVT-associated intermediate phenotypes (i.e. obesity and von Willebrand factor [VWF] levels). Among these variants were a missense variant of CYP4V2 gene (rs13146272) reported to protect from DVT [7] (allele count in DVT cases vs controls of this study: 3 vs 6), a 5'UTR-variant of FGA gene found to increase DVT risk (rs2070011) [25] (allele count in DVT cases vs controls: 12 vs 7) and a variant reported to decrease circulating VWF levels (rs216321) [26] (i.e. expected to have a protective effect since high VWF levels increase DVT risk [27]; allele count in DVT cases vs controls: 0 vs 3).

### Nonsynonymous variation in coagulation genes

Because enhanced coagulation is one of the main pathogenic mechanisms in DVT, particular attention was paid to genetic variation in coagulation factor and anticoagulant genes. A total of 127 variants were identified in 18 genes (*FGA, FGB, FGG, F2, F3, F5, F7, F8, F9, F10, F11, F12, F13A, F13B, PROC, SERPINC1, PROS1, PROZ*). Of these, 41 were missense variants, including 12 novel. Twelve different missense variants were identified in the large *F5 *gene and novel missense variants were found in *FGA *(3)*, F7 *(2)*, F12 *(2), *F5 *(1), *SERPINC1, PROC *(2) and *PROZ*. All missense variants identified in anticoagulant and coagulation factor genes and their functional annotation are summarized in Additional file 1 Table S9. There was no significant difference in the burden of missense variants of coagulation factor genes in DVT cases vs controls, both at single-gene level and at collapsing analysis of all coagulation genes, even when considering in the analysis only variants that were predicted to produce deleterious substitutions by Polyphen 2 or SIFT (see Additional file 1 Table S9 for detailed allele counts). By contrast, DVT cases displayed a higher number of missense variants in anticoagulant genes compared to controls (Table 2; total count of 9 in cases vs 2 in controls, average 0.9 vs 0.18; binomial distribution probability, p = 0.01). Of 5 different missense variants identified in cases, 4 were novel (conversely, only 8/36 variants identified in coagulation factor genes were novel), 3 were predicted to be potentially damaging for protein function by either Polyphen 2 or SIFT and only one (the known SNP rs3024772) was detected also in the control group, suggesting that these missense variants are rare mutations with likely deleterious effect on the encoded proteins.

**Table 2:** Nonsynonymous single nucleotide variants in anticoagulant genes.

Gene	Chromosome	Coordinate	Substitution	Transcript ID	Protein change	dbSNP129	1000 Genomes CEU population, allele frequency	SIFT^a^	Polyphen 2^b^	Alleles cases	Alleles controls
*PROC*	chr2	127895370	C > T	NM_000312	p.R38W	Novel	not present	Dam	Prd	2	0
		127902716	C > A		p.H370Q	Novel	not present	Ben	Ben	1	0

*SERPINC1*	chr1	172150549	G > A	NM_000488	p.P58L	Novel	not present	Dam	Pod	1	0

*PROZ*	chr13	112861006	C > G	NM_003891	p.L11V	Novel	not present	Ben	Ben	3	0
		112874101	G > A		p.R295H	rs3024772	not present	Ben	Prd	2	2

**a **SIFT-based annotation results, Dam indicates that the mutation is predicted to affect protein function (i.e. 'damaging'), Ben indicates that the mutation is predicted to be tolerated (i.e.'benign').

**b **Polyphen 2-based annotation results. Prd indicates that the mutation is predicted to be 'probably damaging', Pod indicates that the mutation is predicted to be 'possibly damaging', Ben indicates that the mutation is predicted to be 'benign'.

### Association analysis of common variants

Nxtgen2plink.rb was used to generate PLINK input files from the BAM and PILEUP files of the 22 individuals that had successfully undergone sequencing. Average SNV-genotyping success was > 90%. Across the 1778 variable sites identified in the target area, we tested 576 variants that had allele frequency of at least 8% and genotyping success > 90% in a case-control association analysis. The top-5 variants were selected for replication (listed in Table 3). Of the 5 variants taken to replication stage 1 in 284 individuals, 2 were sequenced in 719 cases of idiopathic DVT of the lower limbs and 719 controls. The variants that passed stage 1 were a low-frequency missense polymorphism of *PLCG2 *(rs11548656, p.H244R) and a common polymorphism of *FGA *(rs6050, p.T331A). While *PLCG2 *p.H244R had no association after sequencing in > 1400 individuals, *FGA *p.T331A showed a statistically significant association with DVT in a crude association analysis (p = 1.9 × 10^-5^, OR 1.45; 95% CI, 1.22-1.72) and after adjustment for age and gender (p = 0.0081, OR 1.39; 95% CI, 1.10-1.74). PCR and Sanger sequencing also identified 7 missense variants in addition to those targeted at these two loci (6 at *FGA *and 1 at *PLCG2*). There was no association between these variants and DVT at both single-variant and collapsing case-control association analysis. Variant features are summarized in Additional file 1 Table S10. The association of rs6050 with DVT was further tested in multivariable logistic regression models adjusting for several covariates. The association between rs6050 and DVT remained statistically significant after adjustment for age, gender and plasmatic levels of fibrinogen (p = 0.0001, OR 1.39; 95% CI, 1.10-1.74); and for age, gender, fibrinogen levels, body mass index, FVL and prothrombin G20210A (p < 0.0001, OR 1.41; 95% CI, 1.10-1.81). We also tested the interaction (i.e. deviation of the cumulative effect from an additive model) between rs6050 and FVL and between rs6050 and prothrombin G20210A. Both tests were negative (p = 0.44 for interaction with FVL and p = 0.89 for interaction with prothrombin G20210A).

**Table 3:** Common variant association results.

Variant information						Discovery			Replication stage 1 and 2 (combined)						
**Location**	**Substitution**	**Gene**	**Functional annotation**	**Protein**	**dbSNP129**	**Alleles cases**	**Alleles controls**	**p =**	**Effective sample size cases**	**MAF cases, % (n)**	**Effective sample size controls**	**MAF controls, % (n)**	**p=**	**OR**	**95% CI**

chr4:155727040	T > C	FGA	exon - missense	p.T331A	rs6050	10	4	0.004	709	32 (453)	702	22 (312)	1.9 × 10^-5^	1.45	1.22-1.72

chr16:80474413	A > G	PLCG2	exon - missense	p.H244R	rs11548656	4	0	0.013	711	6 (88)	705	6 (83)	0.73	1.05	0.77-1.43

chr4:122837138	T > C	ANXA5	Intron	Na	rs2306416	4	0	0.013	139	15 (41)	138	15 (42)	0.97	0.96	0.60-1.54

chr8:42164111	G > A	PLAT	exon - synonymous	Na	rs1058720	11	3	0.002	139	47 (131)	139	44 (124)	0.61	1.10	0.79-1.54

chr11:47311481	T > C	MYBPC3	Intron	Na	rs11570115	5	0	0.004	137	11 (30)	139	9 (26)	0.63	1.19	0.68-2.07

MAF indicates minor allele frequency; OR, odds ratio; CI, confidence interval; Na, not applicable (the variant does not cause protein sequence changes).

## Discussion

This pilot study is one of the first applications of next-generation sequencing in DVT. Sequencing by oligonucleotide ligation and detection was used to resequence the protein-coding areas of 186 hemostatic/pro-inflammatory genes in cases and controls of DVT. This regional sequencing approach enabled the simultaneous analysis of several dozens of genes in many samples at a fraction of the cost and computation required for whole-exome and whole-genome analysis. At the current level of multiplexing (i.e. up to 64 samples per SOLiD slide, 16 per spot), the analysis of our target area in one sample costs one tenth of a high-coverage exome-sequencing and one hundredth of a high-coverage whole-genome sequencing, with similar proportions in the saving of computational time (3 hours for read mapping vs the few days of whole-exome or the more than 1 week of whole-genome datasets) and information storage-capacity requirements (300 MB per BAM file vs 40 GB per BAM for whole-exome or 400 GB for whole-genome sequencing). For these reasons, regional sequencing is ideal to interrogate relatively small genomic areas deemed of particular functional relevance in a disease. Potential applications of this approach are the sequencing of positional candidate genes or genomic loci identified by genome-wide linkage or association analysis [28], the large-scale replication of the initial findings of exome and whole-genome resequencing studies and the rapid screening of disease-genes in those Mendelian diseases that have several different causal genes [29].

In the field of DVT, the importance of being able to analyze all known hemostatic genes (i.e. the 'hemostateome') has been recently highlighted by Fechtel et al. [30]. The simultaneous sequencing of all hemostatic genes in affected individuals is ideal to study specific combinations of variants in the hemostatic pathway acting in synergy to confer DVT-predisposition. This type of approach has the potential to reveal new disease-predisposing mechanisms, besides the identification of isolated variants that show statistical association with the disease. In this study, the use of molecular barcoding allowed multiplexing without loss of individual sequence information, which is required to fully exploit the potential of sequence data. Sequencing of the 186 genes in 22 individuals yielded more than 1700 genetic variants of different functional type and frequency. Annotation of identified variants revealed several disease-associated variants, a proportion of which had already been reported in association with DVT. Many novel variants with potentially deleterious effect on the function of key hemostatic proteins were also found. These results are consistent with the recent report by Dewey et al. of the genome sequence of a family quartet in which the father had a history of DVT and pulmonary embolism [31]. In the study, the authors identified four different novel nonsynonymous variants in DVT-risk genes and other known thrombophilia associated variants.

In our study, we adopted different analytical approaches to reveal potential associations with the disease at both single-variant and gene/pathway levels. Although the number of analyzed individuals was very small, it was possible to find statistically significant and biologically plausible associations with DVT. An increased burden of rare missense mutations in anticoagulant genes was found in DVT cases compared to controls. Single-variant association analysis followed by replication genotyping in > 1400 individuals identified an association for the rs6050 SNP in *FGA*. The excess of rare missense mutations in anticoagulant genes in patients in whom the deficiencies of natural anticoagulants had been excluded with biochemical assays suggests that a fraction of idiopathic DVT cases might be affected by 'unrecognized' anticoagulant deficiencies, caused by mutations that impart functional effects to which currently used biochemical assays are not sensitive. The association of rs6050, already described by previous candidate SNP studies, was here identified by an agnostic screening of several dozen genes. The association of rs6050 was reported in 4 studies focusing on venous thromboembolism (VTE), therefore including cases of pulmonary embolism without a diagnosis of DVT [25,32-34]. Two of these studies were very small, with total sample size (cases and controls) of less than 500 individuals [25,32]. Ours is the second largest of all the studies on rs6050 both in terms of DVT cases investigated and overall statistical power. Thus, along with previous reports, this study makes of rs6050 one of the most widely replicated variants in DVT. The mechanisms for the association of rs6050 with DVT/VTE are not fully understood. *FGA *rs6050 was reported to result in enhanced coagulation factor XIII-mediated cross-linking of fibrin alpha-chain [35] and to be associated with increased *FGA *transcription [32]. In this report we found no association of *FGA *rs6050 with plasmatic fibrinogen activity, but the fact that rs6050 is a missense SNP and that the amino acid substitution is considered as 'possibly damaging' by Polyphen 2 indeed suggest that the risk for DVT is conferred by an alteration of fibrinogen-alpha chain function.

## Limitations

The approach proposed in this study has limitations. The use of DNA target capture may introduce bias in the detection of genetic variants by allele-selective capture. Both the use of target capture and the short-read length of next-generation sequencing platforms constrain comprehensive analysis of copy number variation (CNV), an important source of genetic variability. However, these limitations can be minimized by increasing sequence coverage and by using complementary genetic analyses (e.g. array-based CNV-ascertainment). Restricting the analysis to a few hundred candidate genes may limit the chance to detect novel associations. On the other hand, the efficiency and cost effectiveness of regional sequencing are ideal for the thorough, large-scale analysis of genetic variation in specific biological pathways such as hemostasis. This approach may provide deep understanding of the mechanisms by which multiple variants in the same biological pathway shape individual predisposition to complex phenotypes.

## Conclusions

Using a molecular-barcode based technique for sample multiplexing on next-generation DNA sequencing platforms we sequenced the coding areas of 186 hemostatic/proinflammatory genes in cases and controls of DVT. We were able to detect known disease-associated variants as well as novel potentially-deleterious variants in disease-associated genes. Our results illustrate the potential of next-generation gene sequencing for the discovery of genetic variation predisposing to common diseases and for the study of inherited thrombophilia.

## Competing interests

The authors declare that they have no competing interests.

## Authors' contributions

LAL, RAG and FP designed the research. LAL, YW, DMM and MW designed the experiments. IM, SMP, EP, MM, FP and PMM gathered samples and clinical information. LAL, DC, MNB, JY and FY analyzed the data. LAL, JY and FY wrote study-specific software. LAL, EP, MM, SES, LLL, HA conducted experiments and handled samples. LAL wrote the bulk of the manuscript. All authors read and approved the final version of the manuscript.

## Pre-publication history

The pre-publication history for this paper can be accessed here:

http://www.biomedcentral.com/1755-8794/5/7/prepub

## Supplementary Material

Additional file 1 **Supplementary Material**. Supplementary Tables and Figures. Click here for file

Additional file 2 **target and tiled regions**. target and tiled genomic coordinate-intervals. Click here for file
