# Supplementary material for: Identification of genetic risk variants for deep vein thrombosis by multiplexed next-generation sequencing of 186 hemostatic/pro-inflammatory genes
Source: BMC Med Genomics. 2012 Feb 21;5:7. doi: 10.1186/1755-8794-5-7 (PMC3305575; doi:10.1186/1755-8794-5-7)
Supplement: Additional file 1 — Supplementary Material. Supplementary Tables and Figures. [file 1755-8794-5-7-S1.DOCX]

**SUPPLEMENTARY MATERIAL**

**INDEX**

**Tables S1-S10 – Page 2**

**Figures S1-S3 – Page 13Table S1.** Target gene list.

| ABO | EDIL3 | HTR1B | NFKB2 | RASGRP2 |
| --- | --- | --- | --- | --- |
| ADAMTS13 | EPS8L2 | HTR1D | NOS3 | RGS7 |
| ADRB1 | F10 | HTR1E | ODZ1 | RND2 |
| ADRB2 | F11 | HTR1F | OS9 | SCARB1 |
| ADRB3 | F12 | HTR2A | P2RY12 | SELE |
| ADRBK1 | F13A1 | HTR2B | PCSK9 | SELP |
| ANXA5 | F13B | HTR2C | PF4 | SERPINB2 |
| APCS | F2 | HTR3A | PKN2 | SERPINC1 |
| APOH | F2RL1 | HTR3B | PKN3 | SERPIND1 |
| ARHGEF1 | F2RL2 | HTR4 | PLA2G6 | SERPINE1 |
| AXL | F2RL3 | HTR6 | PLAT | SERPINF2 |
| C1orf114 | F3 | ICAM1 | PLAU | TACR1 |
| C1QB | F5 | ICAM2 | PLCB1 | TBX2 |
| C1R | F7 | ICAM3 | PLCB2 | TBXAS1 |
| C1S | F8 | ICAM4 | PLCB3 | TFPI |
| CADM1 | F8 | ICAM5 | PLCB4 | THBD |
| CALM1 | F9 | IL17A | PLCG1 | TLN1 |
| CALM2 | FCGR2A | IL1A | PLCG2 | TLR1 |
| CALM3 | FCGR2B | IL1B | PLG | TLR10 |
| CALR | FGA | IL23A | PPP1CA | TLR2 |
| CASP8AP2 | FGA | IL6 | PPP1CB | TLR3 |
| CD34 | FGB | ITFG2 | PPP2CA | TLR4 |
| COL1A1 | FGG | ITGA2 | PPP2CB | TNF |
| COL1A2 | FTO | ITGA2B | PPP3CA | TYK2 |
| COL2A1 | GAS6 | ITGB1 | PPP3CB | TYRO3 |
| COL3A1 | GNAI3 | ITGB2 | PRKCA | VASP |
| COL4A1 | GNAQ | ITGB3 | PRKCD | VHL |
| COL4A2 | GNAS | KNG1 | PRKCQ | VHLL |
| COL4A3 | GNB2L1 | LDLR | PRKD1 | VWF |
| COL4A3BP | GP1BA | LPA | PRKD2 | ZNF544 |
| COL4A4 | GP1BB | LRP1 | PRKD3 |  |
| COL4A5 | GP5 | MARCKS | PROC |  |
| COL4A6 | GP6 | MERTK | PROS1 |  |
| COL6A1 | GP9 | MET | PROZ |  |
| COL6A2 | HIF1A | MFGE8 | PSEN1 |  |
| COL6A3 | HIF3A | MTHFR | PTGS1 |  |
| CR2 | HRH2 | MYBPC3 | PTGS2 |  |
| CRP | HRH2 | NAT8B | PTK2B |  |
| CYP4V2 | HTR1A | NFKB1 | PTX3 |  |

**Table S2.** Characteristics of the individuals who underwent next-generation sequencing.

| ***Individual ID*** | ***Age,***  ***years*** | ***Gender*** | ***Number of thrombotic episodes*** | ***Type of episodes*** | ***Pulmonary embolism at first DVT*** | ***BMI,***  ***kg/m^2^*** | ***ATIII, %*** | ***PC, %*** | ***PS, %*** | ***PT, INR*** | ***aPTT, ratio*** | ***Fibrinogen, mg/dL*** |
| --- | --- | --- | --- | --- | --- | --- | --- | --- | --- | --- | --- | --- |
| DVT_P_01 | 48 | F | 2 | DVT, SVT | No | 30.1 | 120 | 101 | 102 | 1.07 | 0.91 | 361 |
| DVT_P_02 | 20 | M | 2 | DVT (2) | No | 23.3 | 106 | 134 | 188 | 1.04 | 0.81 | 278 |
| DVT_P_03 | 48 | M | 1 | DVT | No | 24.6 | 103 | 116 | 105 | 0.94 | 1.02 | 388 |
| DVT_P_04 | 39 | M | 1 | DVT | Yes | 24.8 | 104 | 77 | 153 | 1 | 1.27 | 301 |
| DVT_P_05 | 35 | M | 1 | DVT | Yes | 24.7 | 111 | 88 | 101 | 1.01 | 0.92 | 279 |
| DVT_P_06 | 32 | F | 2 | DVT (2) | No | 19.8 | 97 | 86 | 101 | 1.05 | 0.93 | 324 |
| DVT_P_07 | 48 | M | 1 | DVT | No | 21.5 | 116 | 63 | 100 | 1.03 | 1.13 | 304 |
| DVT_P_08 | 23 | F | 1 | DVT | No | 30.1 | 95 | 81 | 135 | 1.06 | 1.16 | 374 |
| DVT_P_09 | 37 | M | 3 | DVT, SVT (2) | Yes | 34.6 | 87 | 78 | 166 | 1.02 | 0.94 | 365 |
| DVT_P_10 | 55 | F | 3 | DVT, SVT (2) | No | 22.9 | 98 | 88 | 146 | 1 | 0.9 | 265 |
|  |  |  |  |  |  |  |  |  |  |  |  |  |
| DVT_C_01 | 45 | F | / | / | / | 22.7 | 115 | 78 | 98 | 1.01 | 1.00 | 374 |
| DVT_C_02 | 25 | M | / | / | / | 24.4 | 96 | 81 | 116 | 1.15 | 0.97 | 229 |
| DVT_C_03 | 48 | M | / | / | / | 32.2 | 96 | 129 | 135 | 0.98 | 1.02 | 235 |
| DVT_C_04 | 39 | M | / | / | / | 25.3 | 101 | 72 | 100 | 1.10 | 1.14 | 206 |
| DVT_C_05 | 37 | M | / | / | / | 24.2 | 115 | 135 | 145 | 1.00 | 0.99 | 208 |
| DVT_C_06 | 34 | F | / | / | / | 29.6 | 109 | 141 | 121 | 1.03 | 0.96 | 366 |
| DVT_C_07 | 46 | M | / | / | / | 27.1 | 87 | 156 | 123 | 0.92 | 0.98 | 311 |
| DVT_C_08 | 25 | F | / | / | / | 18.7 | 111 | 118 | 97 | 0.97 | 0.97 | 244 |
| DVT_C_09 | 40 | M | / | / | / | 28.1 | 106 | 113 | 124 | 0.92 | 1.01 | 207 |
| DVT_C_10 | 56 | F | / | / | / | 21.5 | 103 | 89 | 99 | 1.02 | 0.98 | 285 |
| DVT_C_11 | 48 | F | / | / | / | 26.2 | 93 | 110 | 108 | 0.90 | 1.00 | 371 |
| DVT_C_12 | 50 | F | / | / | / | 22.0 | 102 | 100 | 95 | 0.97 | 1.06 | 272 |

Type of episodes reports the type of thrombotic episode patient’s history was positive for, in parentheses is reported the number of episode of that type. DVT indicates deep vein thrombosis; SVT, superficial vein thrombosis; BMI, body mass index; ATIII, antithrombin; PC, protein C; PS, protein S; PT, prothrombin time; INR, international normalized ratio; aPTT, activated partial thromboplastin time.

**Table S3.** Individual sequence and coverage statistics.

| ***Statistics*** | *DVT_P_01* | *DVT_P_02* | *DVT_P_03* | *DVT_P_04* | *DVT_P_05* | *DVT_P_06* | *DVT_P_07* | *DVT_P_08* | *DVT_P_09* | *DVT_P_10* | *DVT_C_01* | *DVT_C_02* | *DVT_C_03* | *DVT_C_04* | *DVT_C_05* | *DVT_C_06* | *DVT_C_07* | *DVT_C_08* | *DVT_C_09* | *DVT_C_10* | *DVT_C_11* | *DVT_C_12* |
| --- | --- | --- | --- | --- | --- | --- | --- | --- | --- | --- | --- | --- | --- | --- | --- | --- | --- | --- | --- | --- | --- | --- |
| **Raw Mb** | 532 | 592 | 525 | 422 | 538 | 548 | 513 | 471 | 367 | 544 | 472 | 488 | 561 | 471 | 463 | 644 | 472 | 592 | 527 | 748 | 501 | 519 |
| **Unique Mb** | 325 | 361 | 326 | 266 | 210 | 183 | 194 | 147 | 203 | 177 | 328 | 305 | 342 | 293 | 176 | 222 | 176 | 186 | 173 | 230 | 175 | 197 |
| **Unique %** | 61% | 61% | 62% | 63% | 39% | 37% | 40% | 41% | 39% | 39% | 60% | 63% | 61% | 62% | 40% | 36% | 39% | 33% | 34% | 32% | 37% | 39% |
| **On –target** | 7% | 6% | 7% | 7% | 7% | 7% | 7% | 8% | 6% | 7% | 7% | 7% | 7% | 7% | 7% | 6% | 7% | 6% | 6% | 5% | 7% | 7% |
| **Average**  **Coverage** | 62 | 62 | 60 | 53 | 34 | 33 | 31 | 28 | 33 | 32 | 63 | 56 | 62 | 58 | 33 | 38 | 31 | 33 | 28 | 33 | 32 | 34 |
| **1x Cov.** | 99% | 99% | 99% | 98% | 98% | 98% | 98% | 98% | 98% | 98% | 99% | 99% | 99% | 99% | 98% | 98% | 98% | 98% | 99% | 98% | 98% | 98% |
| **10x Cov.** | 96% | 96% | 96% | 95% | 88% | 88% | 87% | 87% | 89% | 88% | 96% | 95% | 96% | 95% | 89% | 91% | 87% | 89% | 85% | 89% | 89% | 90% |
| **20x Cov.** | 93% | 93% | 92% | 91% | 74% | 75% | 70% | 68% | 74% | 73% | 93% | 91% | 93% | 92% | 74% | 80% | 71% | 74% | 66% | 75% | 74% | 76% |
| **40x Cov.** | 83% | 84% | 82% | 76% | 39% | 39% | 33% | 27% | 39% | 35% | 84% | 79% | 83% | 80% | 37% | 48% | 35% | 37% | 26% | 38% | 35% | 40% |

**Table S4.** General sequence and coverage statistics.

| ***Statistics*** | *Average* | *Min* | *Max* |
| --- | --- | --- | --- |
| **Raw Mb** | 523 | 367 | 748 |
| **Unique Mb** | 236 | 147 | 361 |
| **Unique %** | 46% | 32% | 63% |
| **On –target** | 7% | 5% | 8% |
| **Average Coverage** | 42 | 28 | 63 |
| **1x Cov.** | 98% | 98% | 99% |
| **10x Cov.** | 91% | 85% | 96% |
| **20x Cov.** | 80% | 66% | 93% |
| **40x Cov.** | 53% | 26% | 84% |

**Table S5.** Individual single nucleotide variant statistics.

| ***Type of variant*** | *DVT_P_01* | *DVT_P_02* | *DVT_P_03* | *DVT_P_04* | *DVT_P_05* | *DVT_P_06* | *DVT_P_07* | *DVT_P_08* | *DVT_P_09* | *DVT_P_10* | *DVT_C_01* | *DVT_C_02* | *DVT_C_03* | *DVT_C_04* | *DVT_C_05* | *DVT_C_06* | *DVT_C_07* | *DVT_C_08* | *DVT_C_09* | *DVT_C_10* | *DVT_C_11* | *DVT_C_12* |
| --- | --- | --- | --- | --- | --- | --- | --- | --- | --- | --- | --- | --- | --- | --- | --- | --- | --- | --- | --- | --- | --- | --- |
| heterozygous | 367 | 324 | 356 | 326 | 292 | 315 | 254 | 275 | 277 | 264 | 366 | 339 | 359 | 344 | 283 | 273 | 260 | 262 | 267 | 311 | 324 | 272 |
| homozygous | 141 | 187 | 166 | 153 | 138 | 127 | 136 | 143 | 145 | 172 | 178 | 165 | 151 | 164 | 150 | 155 | 154 | 160 | 140 | 157 | 116 | 158 |
| Ratio Het/Hom | 2.60 | 1.73 | 2.14 | 2.13 | 2.12 | 2.48 | 1.87 | 1.92 | 1.91 | 1.53 | 2.06 | 2.05 | 2.38 | 2.10 | 1.89 | 1.76 | 1.69 | 1.64 | 1.91 | 1.98 | 2.79 | 1.72 |
| Non-coding | 258 | 246 | 238 | 233 | 166 | 174 | 164 | 175 | 170 | 188 | 254 | 235 | 248 | 232 | 184 | 194 | 172 | 186 | 178 | 197 | 183 | 178 |
| Coding | 250 | 265 | 284 | 246 | 264 | 268 | 226 | 243 | 252 | 248 | 290 | 269 | 262 | 276 | 249 | 234 | 242 | 236 | 229 | 271 | 257 | 252 |
| syn | 150 | 153 | 159 | 145 | 158 | 156 | 135 | 155 | 153 | 148 | 167 | 170 | 157 | 176 | 146 | 136 | 142 | 139 | 132 | 166 | 154 | 145 |
| nsyn | 100 | 112 | 125 | 101 | 106 | 112 | 91 | 88 | 99 | 100 | 123 | 99 | 105 | 100 | 103 | 98 | 100 | 97 | 97 | 105 | 103 | 107 |
| Ratio S/NS | 1.50 | 1.37 | 1.27 | 1.44 | 1.49 | 1.39 | 1.48 | 1.76 | 1.55 | 1.48 | 1.36 | 1.72 | 1.50 | 1.76 | 1.42 | 1.39 | 1.42 | 1.43 | 1.36 | 1.58 | 1.50 | 1.36 |
| nonsense | 0 | 0 | 1 | 0 | 1 | 0 | 0 | 1 | 1 | 0 | 0 | 0 | 0 | 0 | 1 | 0 | 0 | 0 | 0 | 2 | 1 | 0 |
| dbSNP129 | 469 | 461 | 477 | 429 | 404 | 412 | 366 | 390 | 396 | 406 | 489 | 464 | 461 | 462 | 398 | 404 | 382 | 392 | 387 | 442 | 419 | 405 |
| not in dbSNP129 | 39 | 50 | 45 | 50 | 26 | 30 | 24 | 28 | 26 | 30 | 55 | 40 | 49 | 46 | 35 | 24 | 32 | 30 | 20 | 26 | 21 | 25 |
| %Novel | 8 | 10 | 9 | 10 | 6 | 7 | 6 | 7 | 6 | 7 | 10 | 8 | 10 | 9 | 8 | 6 | 8 | 7 | 5 | 6 | 5 | 6 |
| Ti/Tv | 2.60 | 2.28 | 2.81 | 2.57 | 2.71 | 2.71 | 2.82 | 2.73 | 3.06 | 2.60 | 2.32 | 2.68 | 2.64 | 2.74 | 3.01 | 2.72 | 2.66 | 2.94 | 3.42 | 3.03 | 2.96 | 2.55 |
| TOT SNVs | 508 | 511 | 522 | 479 | 430 | 442 | 390 | 418 | 422 | 436 | 544 | 504 | 510 | 508 | 433 | 428 | 414 | 422 | 407 | 468 | 440 | 430 |

**Table S6.** General single nucleotide variant statistics.

| ***Type of variant*** | *Average* | *Range* |
| --- | --- | --- |
| heterozygous | 305 | 254-367 |
| homozygous | 153 | 116-187 |
| Ratio Het/Hom | 2 | 2-3 |
| Non-coding | 202 | 164-258 |
| Coding | 255 | 226-290 |
| syn | 152 | 132-176 |
| nsyn | 103 | 88-125 |
| Ratio S/NS | 1.48 | 1.27-1.76 |
| nonsense | 0 | 0-2 |
| dbSNP129 | 423 | 366-489 |
| not in dbSNP129 | 34 | 20-55 |
| %Novel | 7 | 5-10 |
| Ti/Tv | 2.75 | 2.28-3.42 |
| TOT SNVs | 458 | 390-544 |

**Table S7.** Indel statistics.

| ***Type of variant*** | ***Average*** | ***Min*** | ***Max*** |
| --- | --- | --- | --- |
|  |  |  |  |
| insertions | 3 | 0 | 7 |
| deletions | 5 | 1 | 10 |
|  |  |  |  |
| homozygous | 1 | 0 | 3 |
| heterozygous | 7 | 2 | 13 |
|  |  |  |  |
| non-coding | 7 | 1 | 14 |
| coding | 1 | 0 | 2 |
|  |  |  |  |
| frameshift | 1 | 0 | 2 |
| in frame | 0.2 | 0 | 1 |
|  |  |  |  |
| total | 8 | 2 | 15 |

**Table S8.** Variants present in human gene mutation database, HGMD®.

| **Chromosome** | **Coordinate** | **Minor**  **Allele** | **Major**  **Allele** | **Gene** | **Functional annotation** | **dbSNP** | **Associated disease** | **Association with thrombotic disease** | **Association with DVT or DVT-associated phenotype** |
| --- | --- | --- | --- | --- | --- | --- | --- | --- | --- |
| chr1 | 55301775 | G | A | PCSK9 | Missense | rs505151 | Atherosclerosis, severity, association with | Yes | No |
| chr1 | 167765599 | C | T | F5 | Missense | rs6030 | Thrombosis ? | Yes | Yes |
| chr1 | 167778379 | C | T | F5 | Missense | rs4524 | Thrombosis, increased risk, association with | Yes | Yes |
| chr1 | 167788473 | C | T | F5 | Missense | novel | Thrombosis ? | Yes | Yes |
| chr1 | 167831970 | A | C | SELP | Missense | rs6133 | Atopy, increased risk, association with | No | No |
| chr1 | 167832937 | C | T | SELP | Missense | novel | Higher platelet SELP measures, association with | No | No |
| chr1 | 195297644 | C | T | F13B | Missense | rs6003 | Myocardial infarction, risk, association with | Yes | No |
| chr1 | 205694316 | C | T | CR2 | Regulatory | rs3813946 | Increased transcriptional activity, association | No | No |
| chr4 | 155731347 | T | C | FGA | Regulatory | rs2070011 | Venous thromboembolism, suscep., association with | Yes | Yes |
| chr4 | 187357205 | C | A | CYP4V2 | Missense | rs13146272 | Deep vein thrombosis, reduced risk, association with | Yes | Yes |
| chr5 | 148186633 | A | G | ADRB2 | Missense | rs1042713 | Asthma, nocturnal, association with | No | No |
| chr5 | 148186666 | G | C | ADRB2 | Missense | rs1042714 | Obesity, association with | No | Yes |
| chr5 | 176769138 | A | G | F12 | Regulatory | rs1801020 | Premature myocardial infarction, association with | No | No |
| chr7 | 93881175 | C | G | COL1A2 | Missense | rs42524 | Intracranial aneurysm, suscept., assoc. with | No | No |
| chr7 | 150327044 | T | G | NOS3 | Missense | rs1799983 | Coronary spasm, association with | Yes | No |

**Table S8.** *(continued)*

| **Chromosome** | **Coordinate** | **Minor**  **Allele** | **Major**  **Allele** | **Gene** | **Functional annotation** | **dbSNP** | **Associated disease** | **Association with thrombotic disease** | **Association with DVT or DVT-associated phenotype** |
| --- | --- | --- | --- | --- | --- | --- | --- | --- | --- |
| chr1 | 55301775 | G | A | PCSK9 | Missense | rs505151 | Atherosclerosis, severity, association with | Yes | No |
| chr1 | 167765599 | C | T | F5 | Missense | rs6030 | Thrombosis ? | Yes | Yes |
| chr1 | 167778379 | C | T | F5 | Missense | rs4524 | Thrombosis, increased risk, association with | Yes | Yes |
| chr1 | 167788473 | C | T | F5 | Missense | novel | Thrombosis ? | Yes | Yes |
| chr1 | 167831970 | A | C | SELP | Missense | rs6133 | Atopy, increased risk, association with | No | No |
| chr1 | 167832937 | C | T | SELP | Missense | novel | Higher platelet SELP measures, association with | No | No |
| chr1 | 195297644 | C | T | F13B | Missense | rs6003 | Myocardial infarction, risk, association with | Yes | No |
| chr1 | 205694316 | C | T | CR2 | Regulatory | rs3813946 | Increased transcriptional activity, association | No | No |
| chr4 | 155731347 | T | C | FGA | Regulatory | rs2070011 | Venous thromboembolism, suscep., association with | Yes | Yes |
| chr4 | 187357205 | C | A | CYP4V2 | Missense | rs13146272 | Deep vein thrombosis, reduced risk, association with | Yes | Yes |
| chr5 | 148186633 | A | G | ADRB2 | Missense | rs1042713 | Asthma, nocturnal, association with | No | No |
| chr5 | 148186666 | G | C | ADRB2 | Missense | rs1042714 | Obesity, association with | No | Yes |
| chr5 | 176769138 | A | G | F12 | Regulatory | rs1801020 | Premature myocardial infarction, association with | No | No |
| chr7 | 93881175 | C | G | COL1A2 | Missense | rs42524 | Intracranial aneurysm, suscept., assoc. with | No | No |
| chr7 | 150327044 | T | G | NOS3 | Missense | rs1799983 | Coronary spasm, association with | Yes | No |

**Table S9.** Nonsynonymous variants in coagulation genes. Annotations and allele counts in the next generation sequencing experiments are reported.

| ***Gene*** | ***Chromosome*** | ***Coordinate*** | ***Reference allele*** | ***Variant allele*** | ***Transcript ID*** | ***Protein change*** | ***dbSNP129*** | ***1000Genomes CEU population, AF*** | ***SIFT*** | ***Polyphen 2*** | ***Alleles cases*** | ***Alleles controls*** |
| --- | --- | --- | --- | --- | --- | --- | --- | --- | --- | --- | --- | --- |
| *FGA* | chr4 | 155726496 | C | T | NM_000508 | p.R512K | novel | not present | Ben | Ben | 0 | 1 |
|  |  | 155726824 | C | T |  | p.A403T | novel | not present | Ben | Ben | 0 | 1 |
|  |  | 155727010 | T | A |  | p.T341S | novel | not present | Ben | Ben | 1 | 0 |
|  |  | 155727040 | T | C |  | p.T331A | rs6050 | 0.217 | Ben | Pod | 9 | 4 |
| *FGB* | chr4 | 155706593 | C | T | NM_005141 | p.P100S | rs2227434 | not present | Dam | Ben | 1 | 1 |
|  |  | 155711209 | G | A |  | p.R478K | rs4220 | 0.225 | Ben | Ben | 3 | 9 |
| *F2* | chr11 | 46701579 | C | T | NM_000506 | p.T165M | rs5896 | 0.083 | Dam | Pod | 1 | 1 |
| *F3* | chr1 | 94768650 | C | T | NM_001993 | p.G281E | rs3789683 | not present | Ben | Ben | 0 | 1 |
| *F5* | chr1 | 167750185 | T | C | NM_000130 | p.D2222G | rs6027 | 0.033 | Dam | Prd | 2 | 4 |
|  |  | 167751391 | A | G |  | p.M2148T | rs9332701 | 0.017 | Dam | Prd | 0 | 1 |
|  |  | 167765599 | T | C |  | p.M1764V | rs6030 | 0.25 | Ben | Ben | 4 | 7 |
|  |  | 167776742 | G | A |  | p.P1404S | rs9332608 | 0.042 | Dam | Ben | 1 | 0 |
|  |  | 167777353 | C | A |  | p.S1200I | novel | not present | Dam | Pod | 1 | 0 |
|  |  | 167778179 | T | C |  | p.K925E | rs6032 | 0.225 | Ben | Ben | 2 | 4 |
|  |  | 167778358 | T | C |  | p.H865R | rs4525 | 0.225 | Ben | Ben | 3 | 4 |
|  |  | 167778379 | T | C |  | p.K858R | rs4524 | 0.225 | Ben | Ben | 3 | 3 |
|  |  | 167778502 | T | G |  | p.N817T | rs6018 | 0.025 | Dam | Ben | 2 | 6 |
|  |  | 167785736 | C | T |  | p.R513K | rs6020 | not present | Ben | Ben | 0 | 1 |
|  |  | 167788477 | A | G |  | p.M413T | rs6033 | 0.033 | Ben | Ben | 2 | 4 |
|  |  | 167808137 | C | G |  | p.D107H | rs6019 | 0.05 | Dam | Ben | 3 | 1 |
| *F7* | chr13 | 112818013 | G | A | NM_000131 | p.G157S | novel | not present | Ben | Prd | 1 | 0 |
|  |  | 112820770 | G | A |  | p.R283Q | novel | not present | Ben | Pod | 0 | 1 |
|  |  | 112821160 | G | A |  | p.R413Q | rs6046 | 0.1 | Ben | Ben | 3 | 1 |

**Table S9.** *(continued)*

| ***Gene*** | ***Chromosome*** | ***Coordinate*** | ***Reference allele*** | ***Variant allele*** | ***Transcript ID*** | ***Protein change*** | ***dbSNP129*** | ***1000Genomes CEU population, AF*** | ***SIFT*** | ***Polyphen 2*** | ***Alleles cases*** | ***Alleles controls*** |
| --- | --- | --- | --- | --- | --- | --- | --- | --- | --- | --- | --- | --- |
| *F8* | chrX | 153811479 | G | C | NM_000132 | p.D1260E | rs1800291 | not available | Ben | Ben | 0 | 2 |
| *F9* | chrX | 138460946 | A | G | NM_000133 | p.T194A | rs6048 | not present | Ben | Ben | 1 | 5 |
| *F12* | chr5 | 176763563 | G | C | NM_000505 | p.P385A | novel | not present | Dam | Prd | 0 | 1 |
|  |  | 176763842 | G | A |  | p.P327S | novel | not present | Ben | Ben | 1 | 0 |
|  |  | 176764432 | C | G |  | p.A207P | rs17876030 | 0.008 | Ben | Pod | 1 | 2 |
|  |  | 176764772 | G | C |  | p.L140V | rs35515200 | 0.017 | Ben | Pod | 1 | 0 |
| *F13A* | chr6 | 6097136 | C | G | NM_000129 | p.E652Q | rs5988 | 0.233 | Ben | Ben | 4 | 5 |
|  |  | 6097139 | C | T |  | p.V651I | rs5987 | 0.042 | Ben | Ben | 0 | 2 |
|  |  | 6119865 | G | A |  | p.P565L | rs5982 | 0.217 | Ben | Ben | 4 | 5 |
|  |  | 6263794 | C | A |  | p.V35L | rs5985 | 0.2 | Ben | Ben | 5 | 4 |
| *F13B* | chr1 | 195292774 | T | A | NM_001994 | p.E388V | rs5991 | not present | Ben | Pod | 1 | 0 |
|  |  | 195292912 | A | G |  | p.I342T | rs17514281 | 0.008 | Dam | Ben | 0 | 1 |
|  |  | 195297644 | C | T |  | p.R115H | rs6003 | 0.058 | Ben | Ben | 1 | 2 |

The 1000Genomes CEU population field reports the annotation of variants in the 1000Genomes database; in case the variant was present, the allele frequency of the variant in the CEU population is reported. In SIFT and Polyphen 2 annotation results, Ben indicates predicted benign; Dam, potentially damaging according to SIFT; Pod, possibly damaging according to Polyphen 2, Prd, probably damaging according to Polyphen 2.

**Table S10.** Novel missense variants identified during replication.

| ***Chromosome*** | ***Coordinate*** | ***Ref Base*** | ***Var Base*** | ***Gene*** | ***Functional effect*** |
| --- | --- | --- | --- | --- | --- |
|  |  |  |  |  |  |
| Chr4 | 155726925 | C | T | *FGA* | p.S369N |
|  | 155726929 | C | T |  | p.G368R |
|  | 155726935 | C | T |  | p.E366K |
|  | 155727054 | G | T |  | p.S326Y |
|  | 155727127 | G | C |  | p.P302A |
|  | 155727156 | G | A |  | p.A292V |
| chr16 | 80474389 | C | T | *PLCG2* | p.P236L |

**Figure S1.** Patient selection flowchart.

**Figure S2.** Nxtgen2plink.rb workflow summary.

**Figure S2.** *(continued)*

**Figure S3.** Representative coverage histograms. Coverage (x-axis) is plotted against number of reads (y-axis).
